# German fishery’s adaptation to historic events, Western Baltic Sea, 1890–1950

**DOI:** 10.1007/s13280-022-01768-2

**Published:** 2022-09-22

**Authors:** Karoline Schacht, Rudi Voss

**Affiliations:** 1 Biodiversity Economics, German Centre for Integrative Biodiversity Research (iDiv), Halle-Jena-Leipzig, Puschstrasse 4, 04103 Leipzig, Germany; 2 WWF Germany, International WWF-Center for Marine Conservation, Moenckebergstr. 27, 20095 Hamburg, Germany; 3 Department of Economics, University of Kiel, Kiel, Germany

**Keywords:** Adaptation, Fisheries, Historic event, Kiel Canal, Social-ecological system SES SEFS, Western Baltic Sea

## Abstract

Marine social-ecological systems (SES) have been providing important cultural, social, and economic services for many centuries. They are, however, increasingly threatened by fast changing environmental, ecological, and socio-economic conditions. As historical marine research is increasingly developing into a multidisciplinary endeavour, it offers outstanding points of departure to analyse historic events and the response and adaptation of the respective SES. Such knowledge helps to inform today’s fisheries management and promotes successful management of changing ecosystems. Here, we compile and analyse historical data (1890–1950) of the German Western Baltic Sea fishery SES. This period is characterised by a series of strong impacts due to political, technological, economic, and ecological changes, such as two world wars, a global economic crisis, and other economic or ecological disasters. In our opinion, potential negative effects of those events were in the past attenuated by the system’s high capacity to adapt. However, most of the fishers´ historic options on how to respond and adapt have recently become no longer available. New threats (e.g. climate change) have emerged instead. We conclude that today’s fisheries management needs to integrate options of adaptation by exhausting all present or future opportunities. Adaptive fisheries management should not only focus on environmental change but need to include socio-economic change as well.

## Introduction

In many parts of the world, marine fisheries socio-ecological systems (SES) assure local livelihood and form the basis of important cultural and economic ecosystem services. However, rapidly changing environmental and socio-economic conditions challenge this, asking the management for a response to avoid an undesired evolution of the ecological basis.

Today’s ecosystem-based (fisheries) management needs to develop a better understanding of the risks for marine SES imposed by different external factors in order to set priorities and to facilitate conservation and adaptation of these important systems (Thurstan et al. 2015). This includes identifying the system’s adaptive capacity and options of response, but also, from a historical point of view, its weaknesses. As historical marine research continues to grow as a multidisciplinary endeavour, it offers promising opportunities to analyse historic events, and to learn about the systems’ dynamics in the past in order to provide information to today’s fisheries management.

**Fig. 1:**
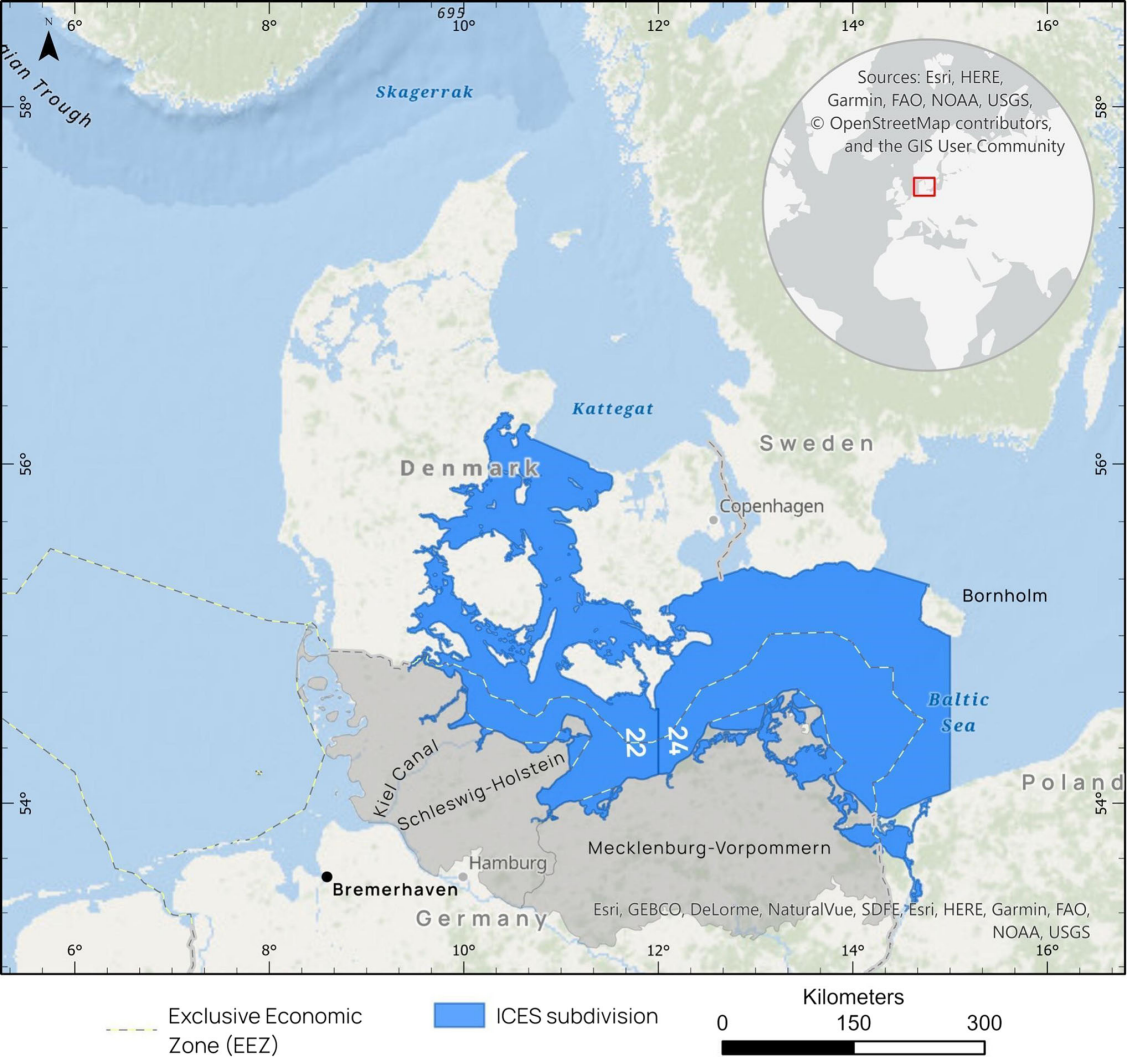
Map of the Baltic coast of Schleswig–Holstein (core area) and neighbouring areas relevant for its fishery over the years. The Western Baltic region (ICES SDs 22 & 24) is highlighted in blue, with the core area of the study indicated by the German Exclusive Economic Zone in SD 22

Here, we focus on the German fishery in Schleswig–Holstein, Western Baltic Sea, as our core area (see Fig. 1): The small-scale fishery in Schleswig–Holstein concentrated in the near shore areas up to ca. 3 nautical miles. The Baltic fishery of Schleswig–Holstein is geographically part of today's ICES Sub-Division (SD) 22. ICES SDs 22 and 24 are often referred to as ‘Western Baltic’ in a management context (highlighted area in Fig. 1). To the north, the Kattegat and the Skagerrak form the transition area to the North Sea. Towards the east of SD 24, the ‘Central Baltic’ commences. The Kiel Canal, which opened in 1895, offers a fast connection to the North Sea. Over the years, the fishery in our core area was connected to and influenced by these neighbouring regions to a variable extent, as technological progress, i.e. intensifying motorization, established possibilities of adaptation for an increasingly mobile fishery.

The Baltic Sea is the world’s largest, semi-enclosed brackish water system. It offers important ecosystem services, including cultural identity, food, recreation, and tourism (Ahtiainen et al. 2019). The western Baltic Sea has a low species diversity compared to many other integral marine systems, and its fishery mainly depends on cod, herring, flatfishes, and sprat.

The Baltic Sea has witnessed several extensive stress factors, such as massive eutrophication, a lack of oxygen, risk of oil spills, marine litter or severely reduced populations of marine mammals due to hunting, contamination, and mass mortalities caused by viruses (Härkönen et al. 2006; BalticStern 2013; HELCOM 2016). These factors may compromise today´s adaptive capacity. Although the situation has slightly improved in some aspects in recent years, e.g. fishing mortality rates are mostly set in accordance with the Maximum Sustainable Yield principle, the important stocks of western Baltic, cod and herring, still remain outside safe biological margins (ICES 2021).

Between 1890 and 1950, a series of unprecedented historical events occurred, which challenged the capacity of adaptation of the local fisheries SES. These events include two world wars, one global economic crisis, and several more regional calamities. Using all accessible historical data, we discuss how selected historical events affected the fishery in Schleswig–Holstein and adjacent regions. We describe whether and how the SES was put to the test by these remarkable incidents and how the fisheries reacted and adapted.

As to historical events, we have taken into account:

- the opening of the Kiel Canal (1895),
- the First World War (WWI, 1914–1918),
- a fishery-induced stock collapse of the Western Baltic plaice stock (starting from 1919),
- a period of economic and political stabilisation (1924–1928) following the end of the German hyperinflation (1923), and the subsequent global economic crisis (1929–1932)
- the Second World War (WWII, 1939–1945) as well as
- a short-term price shock (1949/50).

Our analysis identifies responses to these historical events, aiming to demonstrate fisheries’ former versatility. We hypothesise that adaptation pathways available 100 years ago may not exist any more today. This assumption is not least supported by the overall dire state of the Baltic marine ecosystem as a result of the decades-long overuse. This paper might therefore be a blueprint for historical analyses of other marine SES, which experience social-ecological transformations and where management is challenged in full scope to assist adaptation and conservation of ecosystem services.

We first present a short general overview of the history of the German Western Baltic fishery 1890–1950s, and then, we describe materials and methods used to assess the responses of the fishery to the selected historic events in the selected periods.

## Historical background on the western Baltic Sea fishery

Fishing in the western Baltic Sea has been of subordinate economic importance during the first half of the twentieth century (Eero et al. 2007). At regional scales, its cultural and social significance however is indisputable (Hoffmann 2001), as well as its contribution to food security. Expeditions such as that of the “German Sea Fisheries Association” in 1901 concluded that the Baltic Sea was generally “rather poor in fish” and so contributed to the notion of an economically insignificant sector (Deutscher Seefischerei-Verein 1902). The lack of public fish auctions, the predominant direct marketing, and, ultimately, incomplete statistics on catches, landings, and sales also contributed to the incomplete assessment of the economic rank of the Baltic Sea fishery. Official and comparably detailed records for the North Sea have been available since the late 1890s. In 1927, the German Scientific Commission for Marine Research complained that “an overview of the situation [in the Baltic Sea] is very difficult because of non-availability of reasonably reliable statistics on catches”. (Heinrici 1927).

The Bulletin Statistique from the International Council for the Exploration of the Seas (ICES) started recording fisheries data systematically in 1903. Three years later, the Statistical Yearbook for the German Empire gave a first reference to “fisheries” among other food production activities. The available albeit incomplete data reveal a strong increase in fishing activity: The number of fishers in Schleswig–Holstein, then Prussia’s most northern province, more than doubled between 1894 and 1904, and total landings in German fishery increased sixfold between 1880 and 1910 (see also Table 4).

Future studies might be able to obtain statistics on preceding periods, possibly based on logbooks or export and import statistics, as done for western Baltic herring catches in the period 1200–1650 by Lehmann et al. (2021).

Catch volumes drastically grew during the 1930s when the motorization of fishing vessels sharply increased and more effective fishing techniques like bottom trawls and otter trawls were introduced. Shortly before WWII, Schleswig–Holstein's fishing fleet consisted of around 200 motorized vessels in addition to many open and non-motorized boats (see Table 1). More than half of these vessels had a maximum length of 12 m and a performance of 25 hp, another 85 of them (or 45%) were up to 16 m long with a maximum of 50 hp (Annual reports on the German fishery).

**Table 1:** Length class structure of the Schleswig–Holstein fishery between 1938 and 1957. Source: Annual report of the fishery administration Schleswig–Holstein 1953 and 1957; Bluhm 1952

Year	Total number of vessels	Length < 12 m	12–16 m	16–18 m	> 18 m
1938	188	101 (54%)	85 (45%)	1 (0.5%)	1 (0.5%)
1946	386	185 (48%)	185 (48%)	10 (2.5%)	6 (1.5%)
1953	483	134 (28%)	203 (42%)	65 (13%)	81 (17%)
1957	500	52 (10,4%)	184 (37%)	67 (13%)	131 (26%)

## Materials and methods

**Fig. 2:**
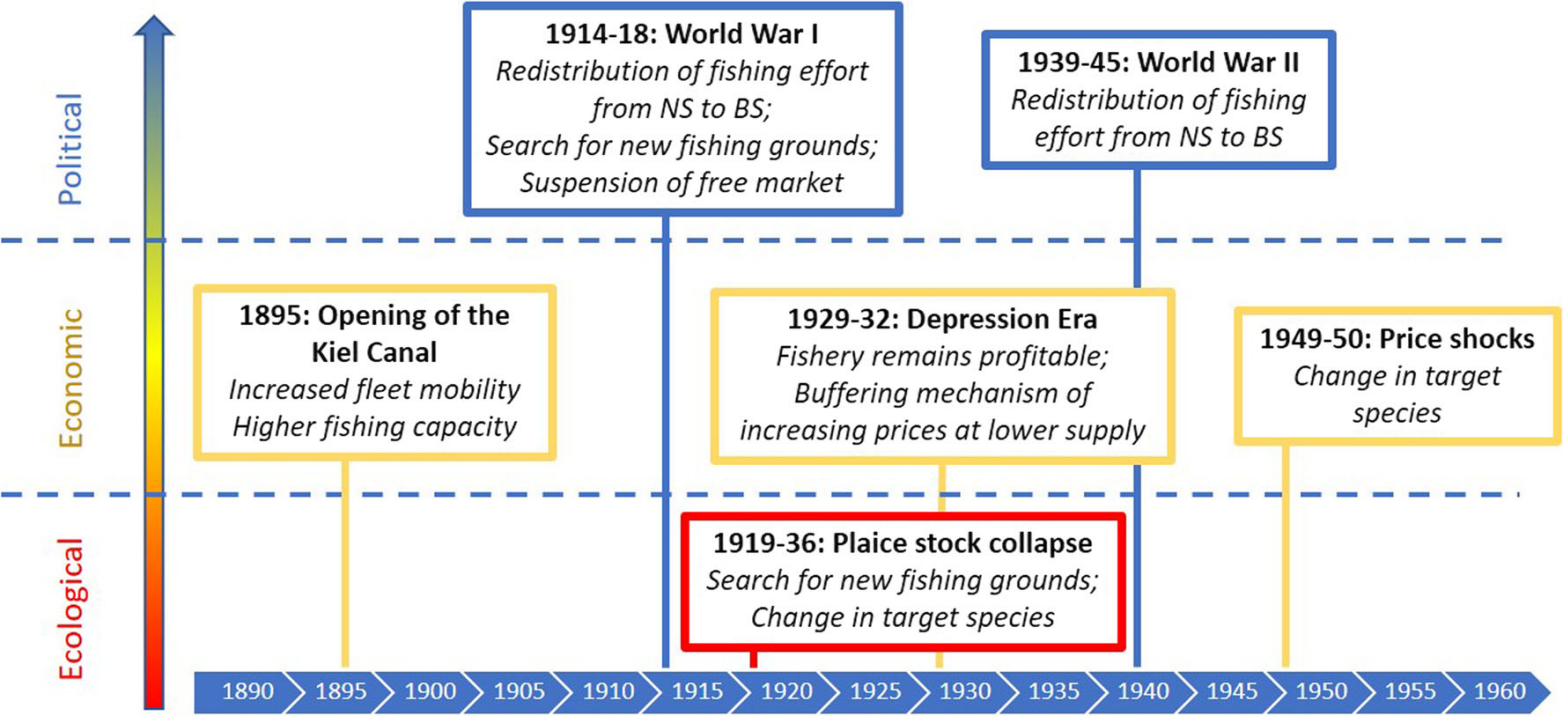
Selection of historic events with impact on the German Baltic fishery in Schleswig–Holstein on three major levels, 1890–1950

In this paper, we focus on the core area of the Baltic coast of Schleswig–Holstein (see also Fig. 1) and consider the timespan between 1890 and 1950. As our study region is functionally connected with its neighbouring areas, and as time series are sometimes only available for broader geographical scopes, we include descriptions of a wider spatial scale wherever informative or necessary (Fig. 2). The available official data for the referenced period of time are partly fragmented but seemed reliable enough to study the reaction of the fishery to the selected historic events. Although we use quantitative records of e.g. fisheries landings, our analysis is to be seen as a qualitative evaluation of time series and historical events. To cross-check our selection of the most influential events for the fishery in that period, we additionally interviewed two representatives of fishery-related museum societies as local informants.

### Local informants

We used semi-structured interviews (see questionnaire below), specifying the period and question of interest, to consult Katharina Mahrt from Alte Fischräucherei Eckernförde e.V. (Old fish smokehouse association, Eckernfoerde) and Uwe Sturm from Verein Museumshafen Probstei e.V. (Museum harbour association, Probstei).

They were able to comment on and describe how historic events affected local fishing businesses. Both emphasised the role of the two world wars, post-war adaptations as well as ecological and economic disruption in the 1920s-30 s, which we accordingly considered in our analysis.

Questionnaire used in semi-structured interviews with two local informants. Interviews have been recorded between October and November 2021 (Box 1).

Box 1 Questionnaire on major changes in the fisheries of Schleswig-Holstein ~1900–1970 **GOAL I**

Designation of periods of substantial change in the fisheries of Schleswig-Holstein, ca. 1900–1970.

**GOAL II**

Identification of driving forces and responses within the social-ecological system.

**I INTRODUCTION**

Why are you personally interested in the fishery in S-H?

- Individual background
- Interests
- Expertise

**II MAIN PART**

Open discussion with leading questions

We are particularly interested in periods when fishing in S-H has undergone major changes as well as in reasons which may, in your opinion, led to these changes. We would like to start at the beginning of the 20th century.

- What did fishing look like then?
- When did the substantial changes occur?

**GUIDING QUESTIONS:**

- Have there been ecological cuts? What did they lead to?
- What about significant technical changes? Which were there? What did they lead to?
- Have there been significant social changes? Which were there? What did they lead to?

**PERIODS TO BE COVERED:**

- First World War 1914-1918
- World Economic Crisis ~1920s
- Second World War 1939-1945

**III CONCLUSION**

Is there anything else we haven´t covered that you would like to add?

Do you have any questions for us?

### Data sources

We evaluated historical sources:

- A daily job diary of Fiete Daniel, a fisherman from Eckernfoerde. Between 1918 and 1923, he kept a diary with detailed descriptions of his day-to-day life as a fisher providing anecdotal evidence also for some of the historical events referred to in this paper.
- Der Fischer-Bote, Norddeutsche Fischerei-Zeitung (Fishers Herald, Northern German Fishery Newspaper), 1910–1940.
  - A newspaper-style publication which provided most recent information on developments at auction places for its target group, e.g. when vessels were up for sale or crews were wanted for hire. This publication was less number driven but packed with anecdotal evidence instead.
- Statistische Jahrbücher für das Deutsche Reich und die Bundesrepublik Deutschland (Statistical yearbooks for the German Empire and the Federal Republic of Germany), 1890–1941 and 1949ff.
  These yearbooks used to be a thorough and detailed data source until WWII. The long list of species in the “landings/value-tables” even distinguished between different qualities, i.e. size classes of the fish.
- Deutsche Fischerei-Zeitung (German Fishery newspaper), 1885–1924.
  When the fish markets—which were at the landing ports—grew along the German coast and were turned into auction platforms, interest in the development of fish prices arose. This weekly newspaper cited both auction and customer prices for selected fish species.
- Jahresberichte über die Deutsche Fischerei (Annual Reports on the German Fishery), 1913–1962.
  This publication is presumably the most detailed one throughout the whole array of historical sources. The annual reports were rather textbooks than mere “reports” and described the development of the coastal fishing business in long paragraphs. They regularly included tables with detailed information on landings, sales revenues, fleet sizes, etc.
- Jahresberichte des Fischereiamtes Schleswig–Holstein (Annual Reports of the Fishery Administration Schleswig–Holstein), 1949–1960.
  These official annual reports concentrated on the Schleswig–Holstein fishery only. Fleet sizes and total landings were reported on a regular basis, and additionally, the reports provide in-depth descriptions of other thematic focal points. Sadly, the time series was interrupted, because several volumes got lost after the war.
- ICES—Bulletin Statistique, landing statistics 1903–1949 and 1950–2010.
  The International Council for the Exploration of the Seas was founded in 1902. It maintained one of the most comprehensive historic databases—the Bulletin Statistique—on fisheries landings data.

All data we used were part of official reports and publications. For this paper, we selected data from the above-mentioned sources and combined them into time series. These series helped to uncover certain changes and their triggers as well as to classify significant transformation processes in the past. We concentrated on cod and plaice as relevant fish species for the fishing sector covered by sufficient data, which were not available for the commercially important herring fishery.

### Units of measure

The historical sources contain information on monthly or annual fish catch volumes measured by the quantities landed (and usually auctioned)—in pounds, kilograms, quintals, or tons. To secure comparability with more recent information, all quantities have been converted into tons, unless otherwise stated.

The results concerning fishery catches during the first World War display the Catch Per Unit of Effort (CPUE) as a relatively simple measure of fish stock size, which is also of economic importance. It was measured as the total catch of fish in tons per day at sea. The CPUE can be influenced by technological progress and by the behaviour of fish and fishers alike, so that time series of stock size estimates can be biased.

The currency-related information is diverse: Within the eighty years between 1871 (establishment of the German Empire) and 1949 (foundation of the Federal Republic of Germany), there were three real currency reforms, and periods of severe inflation.

The only available source for indexing the price data is the seminal work of Hoffmann (1959), which was, however, recently challenged. Data need to be re-checked and corrected, a task currently undertaken by the University of Münster, Germany. As so far, no reliable data basis is available, we did not correct price and revenue data for inflation.

## Results

The historic events analysed in this study spread over 6 decades, and comprise important political, economic, and ecological examples (Fig. 2). Each event and its impacts on the fishery are described in detail below.

### Opening of the Kiel Canal, 1895

**Fig. 3:**
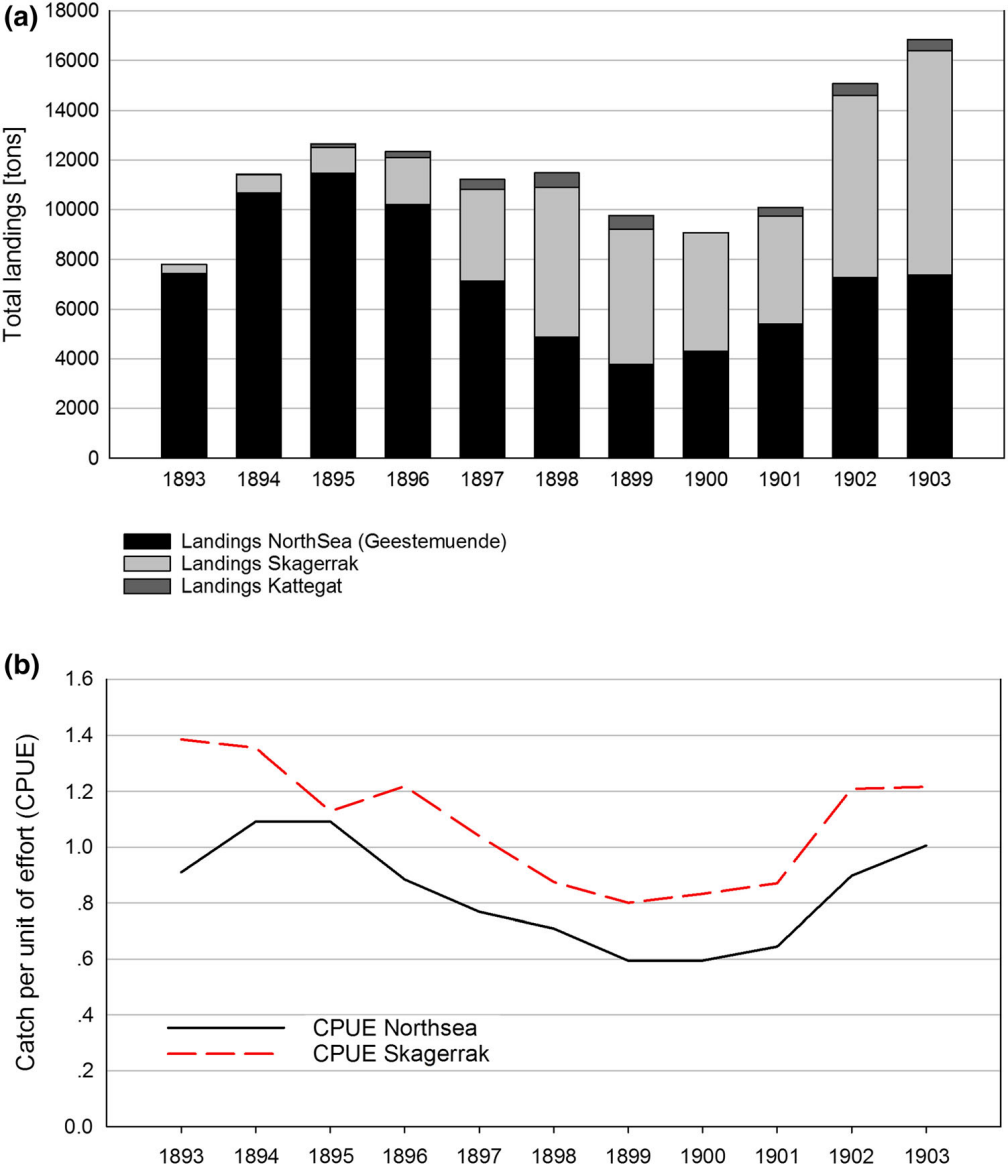
a and b: Reaction of the fishing system to the opening of the Kiel Canal in 1895. 2a: Landings at Geestemuende port (Bremerhaven) from the North Sea (black) and from the Baltic (Skagerrak (light grey)/Kattegat (dark grey)) between 1893 and 1903, 2b: Catch per unit of effort (CPUE) for fishing grounds in the North Sea and the Skagerrak, 1893–1903. Source: Annual Reports for the German Fishery 1893–1903, own illustration

On June 20, 1895, a major construction project of the German Empire was completed: the Kiel Canal (see Fig. 1). Since then, it connects the North Sea and the Baltic coast of Schleswig–Holstein. Fishers on both sides were now able to embark upon checking out new fishing grounds or fish markets without risking the time-consuming and often dangerous detour via Skagerrak and Kattegat (Hansa 1885). Protagonists from both areas could decide more easily which fishing area was more promising, and they took advantage of the new shortcut into the Baltic. The actual relevance of the canal for the fishery is documented by a report dating back to the year 1900: “*They [the fishers] are constantly looking for richer, if more remote, fishing grounds. They visited the Kattegat and the Skagerrak quite a lot, often using the Kaiser-Wilhelm-Canal*^1^* to shorten the route*^2^”. The report gives account of several tenuous years for the North Sea fishers, who finally had to adapt to the situation by searching for and travelling to fishing grounds which were richer in fish compared to the overfished North Sea grounds. Although quantifiable evidence for fishers making use of this new opportunity is missing for the Baltic side of the Canal, it exists for the North Sea: Fig. 3a shows the development of the total landing volumes for the Geestemuende (Bremerhaven, North Sea) fish market between 1893 and 1903. Since the middle of the nineteenth century, Geestemuende had been the most important sea fish landing port and auction place in Germany (AFC and Cofad 2014). Directly after the opening of the canal, landings from the Kattegat and Skagerrak regions significantly increased, while at the same time those originating from the North Sea area sharply dropped. This may point at a temporal shift of fishing efforts from the North Sea into the Baltic and an increase of competition for Baltic fish during this period of time. Baltic catches are reported to come from the Kattegat and Skagerrak regions, and not from the Western or Central Baltic. This might be so due to better catch options, e.g. higher stock sizes in those regions as compared to other Baltic regions.

A relatively simple measure of fish stock size, which is also of economic importance, is the Catch Per Unit Of Effort (CPUE) which is displayed for the three regions in Fig. 3b. It was measured as total catch of fish in tons per day at sea and reported in Statistische Jahrbücher für das Deutsche Reich (Statistical yearbooks for the German Empire and the Federal Republic of Germany), 1890–1903. While applying this coarse measure during this period of major technological change (see below) might complicate any quantitative stock size estimation, useful insight might still be gained from the relative development over the years. Before 1895, the dynamics from both areas appeared to originate from two separate systems. Since 1895, however, the CPUEs of the North Sea and the Skagerrak show an almost parallel course, suggesting that the Kiel Canal turned two fishing areas into one corresponding sea region.

The opening of the Kiel Canal was just one in a series of major changes in the fishing industry. Up to the end of the nineteenth century, the low level of motorization and a correspondingly low fishing capacity were the main reasons for the lower catches in the Baltic Sea, as compared to the North Sea. Since then, motorization and increased engine performance led to greater mobility of the fishing fleets. Our interviewee Uwe Sturm said “Die Motorisierung der Boote zu Beginn des 20. Jahrhunderts veränderte die Fangmöglichkeiten natürlich erheblich. Die Fischer konnten mehr fangen und weiter weg fischen. Aber da Motor und Benzin teuer waren, ging das relativ langsam voran.^3^“

Eero et al. (2007) described a rather slow motorization process until the 1920's in the German Baltic fishery compared to Scandinavian countries. As long as fishing grounds were close to shore and catches were sufficient before WWI (Meyer 1947), there was a lack of interest in motorizing vessels. This could have supported the decision of North Sea fishers to change fishing grounds, it may, however, also indicate, how bad the situation had become by then for fishers and fish stocks in the North Sea.

Technological development enabled the introduction of new and more effective fishing techniques, and the ever-higher CPUE for the Skagerrak reveals that it became indeed worthwhile for a more mobile fleet to sail to richer fishing grounds, in this case from their North Sea home ports to the Baltic regions. For these players, the channel significantly enlarged their available fishing grounds.

**Fig. 4:**
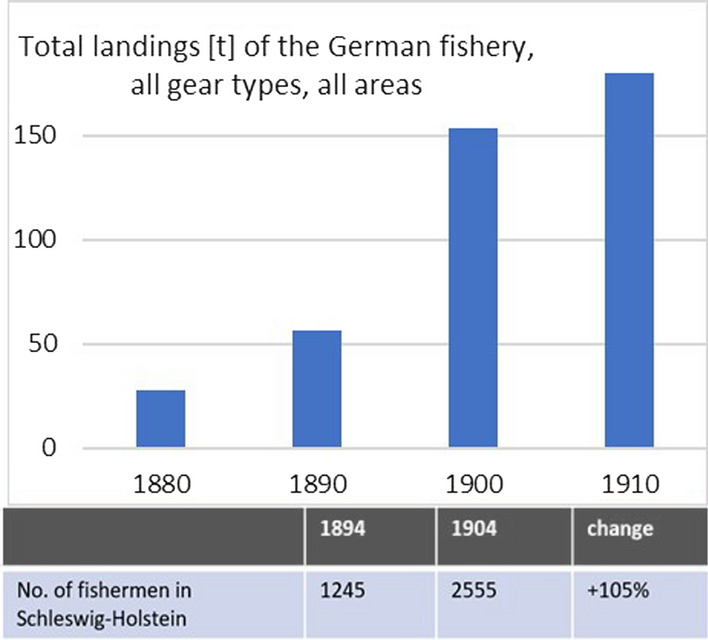
Total landings of the German fishery between 1880 and 1910 (in ´000 tons) and development of the number of fishers in Schleswig–Holstein from 1894 to 1904

The system seemed very dynamic, and additional fishing capacities were set up in a very short time. Within ten years (1894 to 1904), the number of active fishers in Schleswig–Holstein doubled. Figure 4 reveals that total landings of the German fishery (North Sea and Baltic catches) increased sixfold from 28 000 to over 180 000 tons within 30 years. This marks a sharp increase of fishing pressure, which is most probably linked to increased demand and technological progress. In addition to improved mobility of the fleet, the construction of the Kiel Canal caused unintended, but positive side effects, such as the creation of new spawning grounds for Baltic herring in the entire area of the canal, which are retained up to this day (Schimmler 2007).

### First World War, 1914–1918

As soon as the First World War began in 1914, the vibrant German fishery in the North Sea was more or less shut down. Far-reaching trade barriers inhibited almost all trade activities of the German Empire outside the Baltic Sea (Fisser 1990). Several vessel crews were caught unawares by the outbreak of the war in foreign ports, their vessels were confiscated on site and they were taken prisoners of war. Other fishing trawlers were put into service by the Navy. As a reaction to insufficient landings and imports, fish prices rose immensely.

By that time the “Kriegsernährungsamt” (“war food office”) and a central purchasing company (Z.E.G.) as its “spin-off” were founded in 1915 by the German Empire and some of its federal states. Although supervised by the office of internal affairs, the Z.E.G. was run as a commercial company and not as an authority. Its main task was to increase imports of food from (neutral) foreign countries as well as to optimise fish supply and to help prevent speculation. The goods it bought were passed on to local authorities rather than to consumers or retailers. But once food shortage became obvious in autumn 1915, the Z.E.G. introduced a planned economy. This measure temporarily suspended the free market for fisheries (Heidbrink 2003). General fishing strategies remained largely unchanged, with plaice and herring as major target species (Daniel 1923). The fishery was, however, operated by the old and the very young men, who did not have to join the German army. Although illegal, a vibrant black market and direct marketing in the ports evolved at least in some places, circumventing central purchasing.

**Fig. 5:**
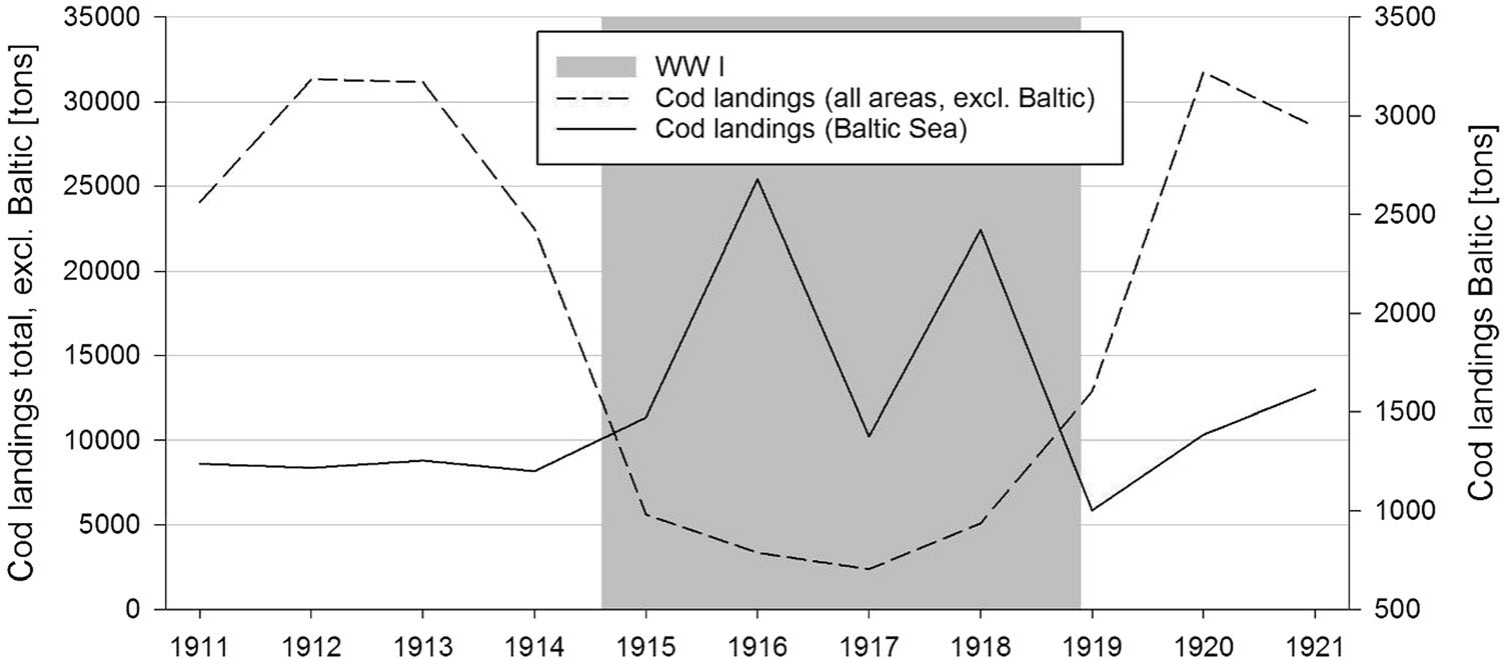
Development of cod landings of the German fishery in the years before, during and after the First World War. Dashed line: cod landings of the German fishery in all areas without the Baltic Sea, y-axis left; solid line: cod landings only in the Baltic Sea (ICES areas IIIb, c and d), y-axis right. Source: Bulletin Statistique 1903–1949, ICES

The Versailles Peace Treaty of 1919 stipulated that Germany had to surrender 40 vessels, of which several were converted into minesweepers. The ones under construction had to be completed at the dockyards and then handed over to the victors as well (Fisser 1990). These arbitrations determine the development of German cod landings around the years 1911–1921 (Fig. 5). While the trade blockades in the North Sea are reflected as a forced drop in the catches, the landings for cod in the Baltic Sea rose to a new high, especially in the year 1916.

Right after the war in 1918, the revival of fishery back to pre-war levels was seemingly easy to achieve, landings from the North Sea rose quite steeply and the system seems to return to its original state. Probably, the forced break for almost all fishing activities during wartime let stocks recover and contributed to the observation that pre-war levels were resumed already in 1920.

### Collapse of plaice stock, starting 1919

**Fig. 6:**
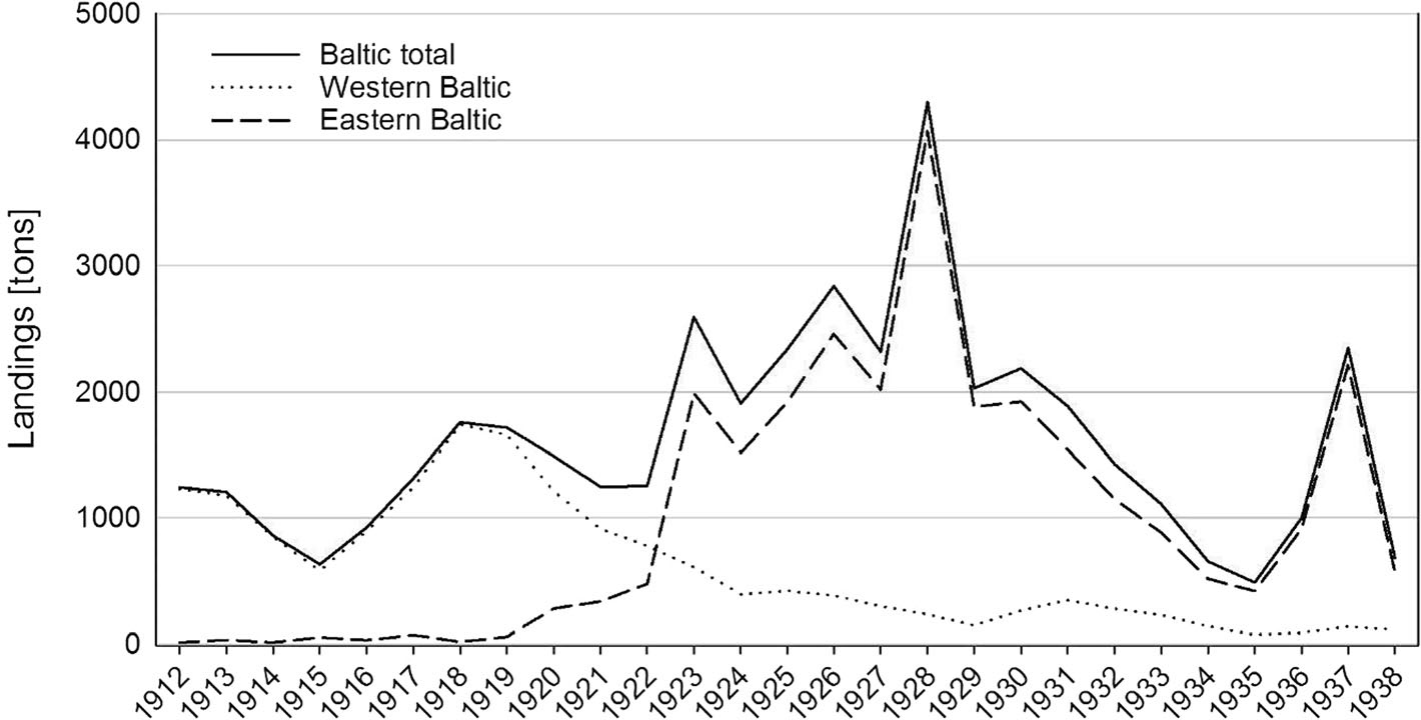
Plaice landings of the German Fishery 1912–1938, solid line: total Baltic landings, dashed line: only eastern Baltic, dotted line: only western* Baltic, all in tons. Source: Statistical Yearbook for the German Reich; Fischer 1939. * line was drawn at Ahrenshoop (Pomerania, see also Fischer 1939)

In the beginning of the twentieth century, in Schleswig–Holstein, flatfish fishery was even more important than cod fishery. Especially during WWI, the German fleet fished heavily for flatfish, including plaice, obviously driven by a severe general food shortage. Starting in 1919, the plaice catches began to dwindle in German waters in the Western Baltic (Fig. 6), as the fishery had reduced the stock down to “the demise of nearly nothing” (Heincke and Mielck 1925; Fischer 1939). For many years, the fishing grounds off the Schleswig–Holstein coast did not recover and did not allow for relevant plaice yields anymore. “Überfischung ist kein so neues Problem. Das schaffte die Fischerei damals [in den 1920er Jahren] auch schon. Der Rückgang des Schollenbestandes ist da ein gutes Beispiel“,^4^ Uwe Sturm said.

Finally acknowledging the adverse situation, in March 1936, the German Research Foundation granted 1.500 Reichsmark to the scientist Erich Fischer in order to explicitly study the reasons of the “catastrophic decline” of plaice catches in the Kiel bight (DFG Archive). He concluded that the combination of several weak year-classes with a growing fishing effort caused the sharp decline amplified by a shift in length distribution and maturity-at-age. Fischer asked for strict regulations to protect any future-rich year-class to come so that the stock may rebuild itself and become of lasting value to the fishery (Fischer 1939).

During this decline of the fishery in Schleswig–Holstein and the Western Baltic, plaice catches remained high in the areas east of Bornholm and along the Swedish coast. Therefore, several German fishers from Schleswig–Holstein adapted to the situation by moving their home ports further east, to Mecklenburg and Pomerania, to reach those fishing grounds more easily, as anecdotal evidence shows, found in the working diary of a fisher from Eckernfoerde, Schleswig–Holstein (Daniel 1923).

Up to 1922, loss of volume in the Western Baltic could not fully be compensated by fishing activities in eastern areas, and German total plaice catches kept decreasing in total (Fig. 6). From 1923 onwards, however, plaice catches from the eastern Baltic area grew enormously (Fig. 6). When the plaice catches finally began to drop in 1930, the fishery changed the main target species again, this time to flounder. The steep increase of flatfish catches towards the mid-1920s was the result of a short “bonanza” on a newly discovered, almost unfished resource in combination with a period of flatfish peak abundance, as documented by Tomczak et al. (2022) for the years 1925–1936 in the central Baltic Sea. This period ended by a potential shift of regime, including the increasing relative importance of cod within the system.

### Depression era, 1929–1932

War had made fishing an incredibly expensive business. According to calculations by various sources, the operating costs for fish steamers grew more than twelve times between 1913 and 1920 (a.o. Annual reports on the German Fishery).

**Fig. 7:**
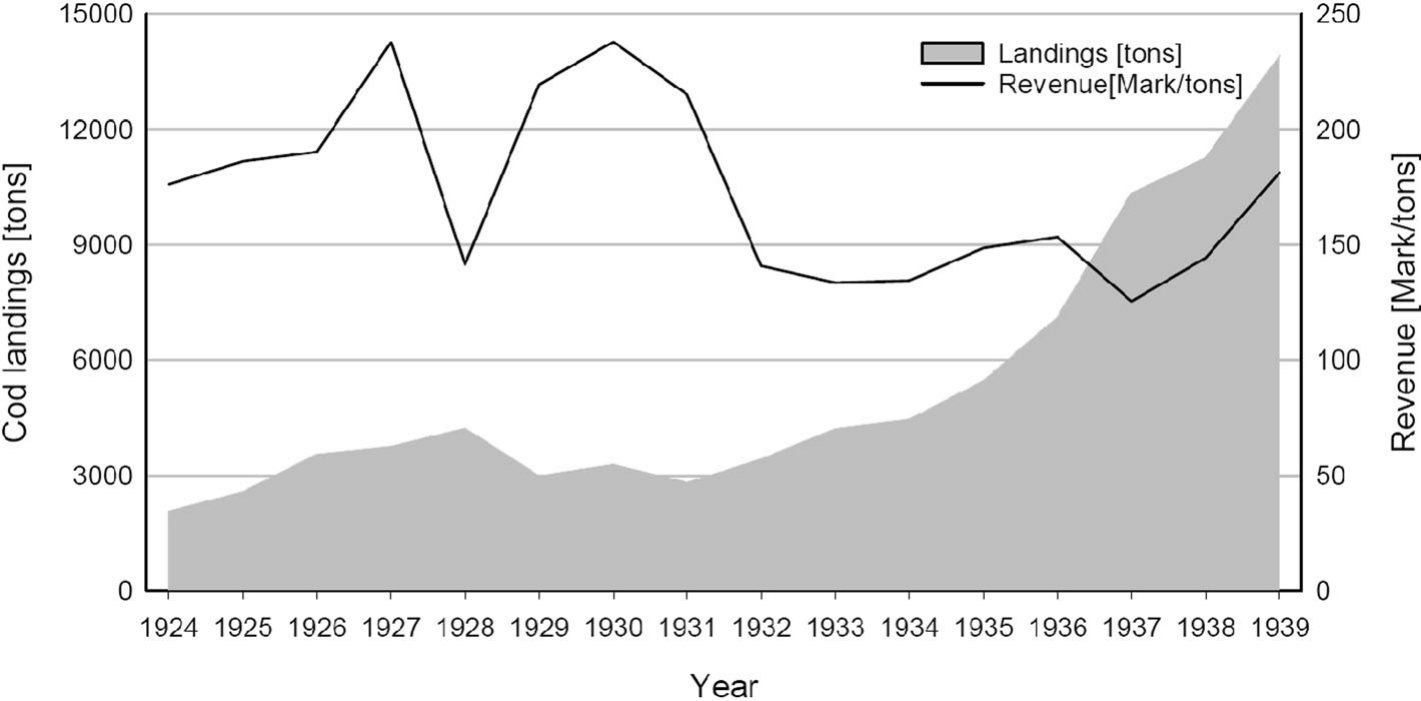
Landings (grey area) and revenues (solid line) of the German Baltic cod fishery, 1924–1939. Source: Statistical Yearbook of the German Reich, Yearbook on the German fishery, own calculations

After years of high and increasing inflation at the beginning of the 1920s, the German hyperinflation ended in 1923, shortly before the introduction of the “Reichsmark” in 1924. The years 1924–1928 were a period of economic and political stabilisation in Germany. Figure 7 shows that Baltic cod landings more than doubled from 2000 to over 4000 tons in this period. With the onset of the global crisis in 1929, which climaxed in Germany in winter 1931/32, cod catches declined. Revenues, however, remained high or even increased, which must be put in relation to changes in cod prices. As the consumers´ price index stayed rather stable in 1924–1931, we can assume that German fisheries (in Schleswig–Holstein and the Baltic) remained profitable, even in times of a world economic crisis.

The year 1933 brought Hitler’s seizure of power. While cod landings did not reach pre-crisis levels until the mid-1930s, they showed a pronounced increase to around 14 000 tons in 1939, shortly before WWII. This steep increase mirrors the fact that Nazi ideologues perceived fish an inexhaustible source of animal protein (Pelzer-Reith and Reith 2009), which should contribute to fill the “protein gap” in the German population’s nutrition.

Compared to other industries, but also in comparison to other nations, the fisheries system of the Western Baltic Sea appeared rather self-contained and stable (in terms of catch quantity and revenues) during the Great Depression. For example, the international fish trade from Iceland and Norway crashed completely in the same period, as prices dropped to levels that did not even cover freight costs (Jonsson 2009).

### Second World War, 1939–1945

**Fig. 8:**
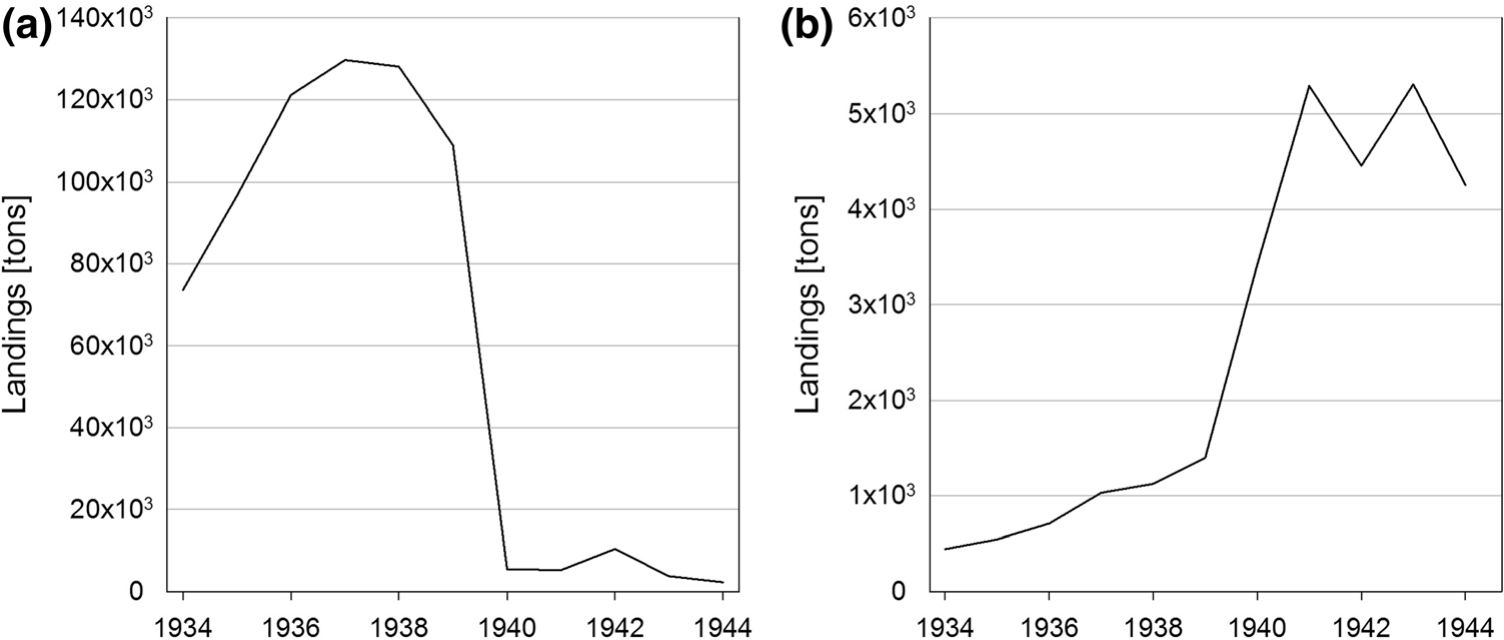
a and b: German cod landings 1934–1944. **a** All areas except Baltic Sea, **b** Baltic Sea only. Source: Annual Reports on the German Fishery

The years before WWII were quite profitable for the fishery. 1938 is considered to be the most successful year in the history of German fishery until the mid-1950s (Statistical Yearbook for the FRG 1953). This is partly attributed to the measures the Nazi government took to deal with the protein shortage by expanding high-sea fishery, by reducing the need for imports and by increasing fish consumption over the course of a Four-Year plan (Pelzer-Reith and Reith 2009). We illustrate the overall development by concentrating our data evaluation on the cod fishery: The outbreak of war caused a collapse of the North Sea high-sea cod fishery (Fig. 8a) as fishing in the North Sea became impossible during the war. This required restructuring and adaptation, and so parts of the North Sea fleet moved into the Baltic to exploit the hitherto lightly fished resources (Meyer 1952; Fig. 8b).

The reallocation of fishing power, combined with the increasing importance of the Baltic Sea for the German fishery during the war, is mirrored by an increase of cod landings at the Baltic coast of Schleswig–Holstein (Fig. 8b). Total cod landings rose fourfold between before and after the war. During this period, numerous innovations were put into practice, particularly in terms of net technology.

### Sudden price shock, 1949/1950

At the end of WWII, numerous so-called “refugee fishers” arrived at the coast of Schleswig–Holstein, who were in fact displaced from their West Prussian and East Prussian homeports by the Soviet Red Army. “Gegen Ende und nach dem Zweiten Weltkrieg veränderte sich das Bild der Fischerei bei uns erheblich. Es kamen viele Flüchtlingsfischer mit ihren Booten, die oftmals deutlich größer und leistungsstärker waren als die der einheimischen Fischer.“^5^ (Uwe Sturm, interview). This resulted in a drastic increase in fishing capacity. Bluhm (1952) described the enormous boom in the Baltic fishery of Schleswig–Holstein after 1945: Within five years after the war, the local population of several fishing communities more than doubled and so did the fishing capacity. According to the author, fish became “less and less a scarce commodity and in the end, it was even difficult to sell”.

**Fig. 9:**
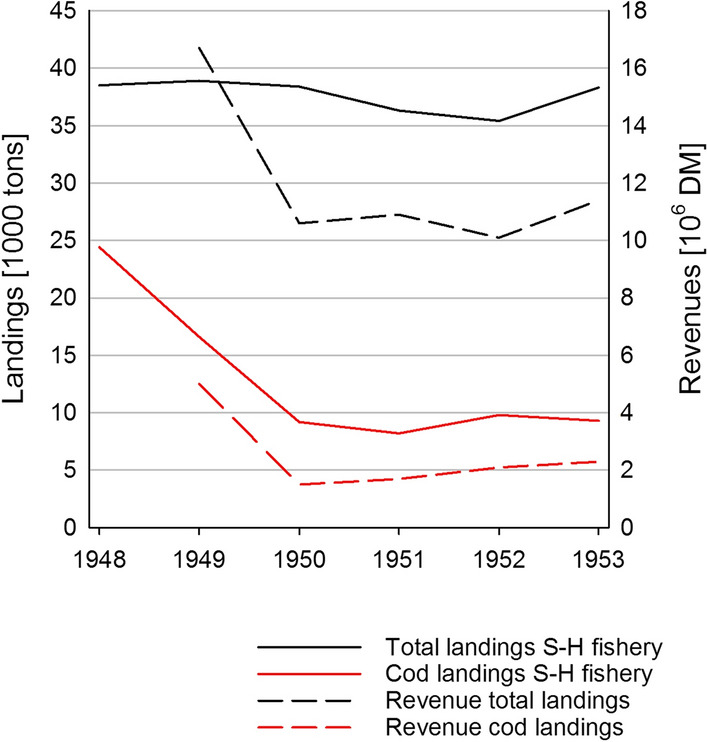
Comparison of total vs. cod landings and revenues of the Schleswig–Holstein fishery between 1948 and 1953. Source: Bluhm 1952; Annual reports of the fishery administration of Schleswig–Holstein, 1954

By the year 1948, the cod fishery in Schleswig–Holstein had acquired major importance and its volumes and values reached a triple of those of the once dominant herring fishery, with two thirds of the total landings in Schleswig–Holstein being cod (Fig. 9). The enormous supply of cod finally led to a sales crisis, which began in the first few months of 1949, when the prices for cod as the main target species started to collapse, presumably because the increased volumes of catch exceeded demand by far. A subsequent drop in prices is documented, and the proportion of cod catches on total landings steeply dropped to less than 50 percent in 1949, continuing to fall to less than 25 percent in 1950 and 1951 (Fig. 9; Annual Report of the Fishery Administration Schleswig–Holstein, 1950–52). The Baltic fishery of Schleswig–Holstein adapted by switching back to herring, so that total catches in the area remained almost constant (Fig. 9). In 1950/51, herring catches were again exceeding those of cod.

Total revenues, however, did not remain constant, but dropped considerably. This points at the importance of cod to the local and regional economy at that time and caused a “*desperate situation*” (Bluhm 1952). The resulting competition drove all those fishers out of business, who were not able to adapt.

## Discussion

Here, we analysed a series of major historical events causing disturbances to the German Baltic fishery in Schleswig–Holstein and described the corresponding changes in its SES. We studied the reaction and adaptation of the fishery system to these primarily socio-economic hazards in order to gain insights for today's management. Generally, a historical perspective on marine SES can help to get a better understanding of today’s state of the system. It may contribute to set achievable goals for its management nowadays and identify suitable measures to reach them (Eero et al. 2007).

### Baltic historical context

We described different trends and developments of the German fishery in the Baltic Sea. We largely built these descriptions on historical data sources. While the quantitative data go back to 1890, some libraries and other archives provide data that date back to the fifteenth century. Poulsen (2007, 2010) provides an assessment of such data leading to “an appraisal of the historical evidence”. Works from different sea areas (Adriatic Sea and Mediterranean: Fortibuoni et al. 2017; Northwest Atlantic: Lear 1998; Netherlands: Bennema and Rjinsdorp 2015), as well as the Baltic (MacKenzie et al. 2002, 2007) mainly focus on the historic variability of the fishery. Other works include time series of country-specific fisheries in the Baltic Sea region (Gulf of Riga: Gaumiga et al. 2007; Swedish Baltic Sea: Ojaveer et al. 2007; Bornholm, Denmark: Bager et al. 2007). The potential adaptation of a fishery to remarkable historic events, as emphasised in this paper, is less often investigated. In any case, it is important to consider whether historic trends might be attributed to data artefacts, including how the data were collected. This study obtained most of the data from official statistical reports on fisheries, where generally no details are given on how these data were acquired in the first place.

### Dealing with crises

The opening of the Kiel Canal altered competition for the Baltic fishery of Schleswig–Holstein, as fleet segments based in the North Sea could now more easily access the Baltic fishing grounds. Higher fishing pressure, putting sustainable exploitation at a risk, resulted from this technological progress. As to the fishery of Schleswig–Holstein and in the Western Baltic Sea, it stipulated an increase in catches. The observed increased share of catches originating from the Western Baltic and landed in Geestemunde (North Sea) might be explained by market competition, as railways readily transported fresh fish from the North Sea to the inland markets—an option not equally available at the Baltic coast. The opening of the Kiel Canal most probably led to a momentary increase of the amount of fish being caught in the Western Baltic. In the long run, however, the local fishery of Schleswig–Holstein was, however, much more influenced by a steadily increasing local demand for fish, especially since once Kiel was made Germany’s most important war port. Fleet exchanges between the Baltic and the North Sea still occur today, with larger vessels occasionally shifting effort from the North Sea to the Baltic, in case quota regulations allow it. As in the beginning of the twentieth century, such exchanges increased competition.

The redistribution of fishing effort from the North Sea into the (Western) Baltic during WWI and WWII was fully driven by external factors, which made German fishing activities in the North Sea almost impossible. In both cases, official landing statistics must be regarded as conservative, as indications for large black-market transactions exist (e.g. Daniel 1923). While the German population suffered from a partly severe food shortage, coastal fishing communities were better off. During WWI, innovative, adaptive fishers were able to find the best fishing grounds and landing places, including ports outside Schleswig–Holstein, and even in foreign countries (Daniel 1923) to secure their income—despite any official planned economy. However, the large cod catches in the Baltic during WWII cannot be explained by fishers´ innovation, increasing fishing effort or technological developments alone. Analysis of national catch, effort and price data of several countries for the main fish species in the Baltic Sea suggest that it is linked to increased abundance in fish (Hammer et al. 2008). One explanation is that in the years before WWII, larger trawlers were banned from the Baltic Sea to protect flatfish stocks, which, from a commercial point of view, were among the most important species by then. Consequently, cod was protected during that time, too, and stocks could recover. Another reason is a shift in the central Baltic ecosystem from a flatfish-dominated state towards a higher importance of cod in the ecosystem (Tomczak et al. 2022). It becomes obvious that historic data must be interpreted with the help of socio-economic data as well as ecological backgrounds.

During the Great Depression, cod prices increased as a reaction to reduced supply. Such mechanisms are typical for less integrated economies (Jacks et al. 2011). The increase in the sales price partly buffered the economic shock, and probably turned the fishery more resilient at that time. The strong external shock could, however, not be fully handled by the market, so that parts of the industry disappeared even though the biological resources were sufficiently productive. If, on the other hand, demand falls short in comparison to realised catches, as observed in 1949/50, supply–demand interaction might as well cause an additional risk.

Today, the German fishery in Schleswig–Holstein as well as in the surrounding Western Baltic Sea seems to be on the brink of a collapse. Stock status and catches of the two main target species for the fishery, cod and herring, declined dramatically over the last decade. Accordingly, the number of active fishing boats decreased by around 50 percent from 1950 to the 1990s (Möllmann et al. 2021). This has considerable impacts, as the small-scale fleet is not only economically important, via direct employment and tourism, but also boasts of a high socio-cultural value for coastal communities.

### Adaptation strategies now and then

The observed historic responses and adaptation options are of particular interest for any lessons to be learnt. They include the three main adaptation strategies to climate change observed in the groundfish trawl communities in the Northeast United States (Papaioannou et al. 2021): *shifting fishing grounds, shifting target species, and shifting port of landing*. Some of the historic responses we observed are no longer available to management: Redirection of the fishing fleet towards so far under-utilised areas, as observed during the stock collapse of plaice, is for no longer possible in the fully exploited Baltic Sea. A suspension of the free market, as done during WWI, also seems highly unrealistic. Furthermore, the buffering mechanism of increasing prices at lower supply is still in existence (Voss et al. 2022) but does not work so efficiently anymore because, in the last decades, fish and fishery products have increasingly become globally traded goods (Ammar et al. 2022). Trade liberalisation and technological advancements were the main drivers of this trend, leading to fish being the globally most traded food product (FAO 2020). Therefore, prices show little reaction to changes in local/regional supply chains nowadays, as they are dominated by the world-market. Indeed, multiple general trends in fisheries worldwide suggest that international trade has begun to influence local governance (e.g. Crona et al. 2016). To cover demand, the EU market has become highly dependent on the import of fishery products (Aranda et al. 2019; EUMOFA 2020). Only if the current EU Common Fisheries Policy, which aims to reduce this dependence, manages to strengthen the EU's own supply in the market, this mechanism of adaptation may in some degree be restored.

A change in target species, observed as a response to price shocks and the plaice stock collapse, is only partly possible in the Western Baltic. Species diversity is generally low here. Flatfish are currently abundant (ICES 2021), but demand is low, and sales cannot fully compensate for the loss in herring and cod fishery. Stock enhancement programs like those initiated by the German government as a response to the plaice stock collapse in the 1930s are expensive and unlikely to be successful for cod and herring. Above all, the global climate crisis is putting marine ecosystems to the test as to structure, function, and services provided. This further underlines that a developed fishery management plays a key role in steering the Western Baltic fishery to a sustainable future.

### Options for management responses to crises

Fisheries management is complicated by the fact that fisheries are embedded in complex SES. In situations, in which uncertainty and change are the key features of the ecological and social landscape, one major management target might be to increase the system’s resilience. Resilience of a SES is defined as the capacity of the system to adapt to change, persist disturbance, learn, self-organise, and trtransform away from unsustainableansform away from unsustainable social-ecological pathways (Folke et al. 2010; Folke 2016). The capacity to adapt is higher, if there is room for “novelty” (Ammar et al. 2022), i.e. when the system is allowed to explore alternative dynamics and structures (Allen and Holling 2010). Adaptation capacity might become an essential asset when all existing pressures on marine ecosystems are exacerbated by effects of the climate crisis.

However, today’s fisheries management and stakeholders often value stability, e.g. maintenance of employment, in contrast to fostering novelty. Stabilising management actions decrease the short-term variability and cause a loss of general SES resilience (Folke et al. 2016). In contrast, allowing for flexibility and species diversification—as observed in the past—buffer against large-scale environmental and market changes and increase the adaptive capacity of fishing communities (Cline et al. 2017).

For example, fostering technological progress related to improved selectivity and reduced bycatch will contribute to reducing the risk of fishery closures. A reduction of the fishing capacity or fishing pressure, as observed during the two world wars and the world economic crisis, seems inevitable. Scientific investigations like those initiated after the plaice stock collapse need to be further intensified for a better understanding of the underlying mechanisms of low stock productivity. Strong management measures to rebuild the fish stocks, like banning certain fleet segments (as observed before WWII), or a complete temporal lock-down of the fishery, including recreational fishing, need to be considered. And finally, it might be necessary to trade elements of stability (e.g. the “relative stability” principle in the EU) against better adaptation possibilities, and therefore higher resilience (Ammar et al. 2022).

Our results demonstrate that there is significant and relevant information available for historic periods to inform recent fisheries management decisions. Our analyses rely on compilations of national landings data from auction places in Germany which have some shortcomings and gaps. However, all available contextual data point in the same direction and thus the general findings seem plausible. We argue that successful management will need to understand the interactions of physical, ecological, and socio-economic drivers of the fishery in order to inform the emerging ecosystem-based fisheries management (EBFM) approach (Karnauskas et al. 2021). Still, a systematic implementation of EBFM remains a challenge, also in the Western Baltic Sea.

## Conclusion

Fostering adaptive capacity and therefore resilience of marine ecosystems and their related fisheries towards an increasingly changing environment should be priority objectives for today’s fisheries management. This is challenged by stakeholders and management who often value stability. The most historic adaptation and response options are no longer at hand, while threats (e.g. climate crisis) have emerged.

We conclude that fisheries management needs to integrate other options of adaptation, using all remaining or emerging opportunities. Adaptive fisheries management should not only focus on environmental change but needs to include socio-economic change as well.

## Footnotes

1 „Kaiser-Wilhelm-Canal” was the Kiel-Canal’s name until 1948.

2 Translated quote: “Sie [die Fischer] suchten daher ständig nach reicheren, wenn auch abgelegeneren Fischgründen. Das Kattegat und Skagerrak besuchten sie oft, meist nutzten sie den Kaiser-Wilhelm-Kanal, um ihre Route zu verkürzen.“ Taken from *Mittheilungen der Section für Küsten- und Hochseefischerei*, 1900.

3 Translated quote: “The motorization of boats at the beginning of the twentieth century changes the fishing opportunities considerably, of course. Fishers could catch more and fish farther away. However, since motors and gasoline were expensive, progress happened only slowly”.

4 Translated quote: “Overfishing is not such a new problem. The fishery already did that back in the 1920s. The decline of the plaice stock is a good example”.

5 Translated quote: “Towards the end and after WWII the image of fishing in our country drastically changed. Numerous refugee fishers arrived with their boats which were much bigger and more powerful than those from resident fishers”.
